# Response to Letter to Editor of Perretti et al. re disorders of arousal and timing of the first period of slow wave sleep: Clinical and forensic implications. Sleep Medicine X 2022:4: 100057

**DOI:** 10.1016/j.sleepx.2023.100062

**Published:** 2023-01-20

**Authors:** Mark R. Pressman

**Affiliations:** Pressman Sleep and Science Forensics, LLC, Ardmore, PA, USA; Lankenau Institute of Medical Research Lankenau Medical Center, Wynnewood, PA, USA; Dept. of Medicine, Sidney Kimmel Medical College, Thomas Jefferson University, Philadelphia, PA, USA; Charles Widger School of Law, Villanova University, USA

**Keywords:** DOA, Disorder of Arousal, LSWS, Latency to Slow Wave Sleep

To the Editor,

Thank you for allowing me to respond to a Letter to the Editor concerning my recent publication in Sleep Medicine X [1]. The authors have presented both unpublished data from a publication of their own [2] as well as from Lopez et al. [3]. Combined they include latency to slow wave sleep (LSWS) data from an additional 258 clinically diagnosed patients with a Disorder of Arousal (DOA) primarily sleepwalking and sleep terrors. They further have presented not just the mean or median LSWS as noted in my previous publication, but the minimum LSWS noted. These minimum LSWSs ranged from 5 to 10 minutes after sleep onset and were recorded in patients who were not sleep deprived or alcohol intoxicated.

The scoring of the LSWS was based on the same methodology for scoring delta EEG waves derived from the original Rechtshaffen and Kales scoring manual [4]. As noted in my publication this method requires a peak-to-peak amplitude of 75uv for scoring of delta EEG. As discussed in the manual and in my publication this criterion has no basis in empirically derived sleep science and is essentially arbitrary. Further, scoring of delta EEG and SWS sleep has been found to be unreliable between qualified scorers and sophisticated sleep laboratories [5]. A reduction in the amplitude criteria from 75 uv would likely result in earlier onset of SWS sleep. Episodes of DOA have also been reported in N2 sleep as well suggesting LSWS shorter than 5–10 minutes might also be possible with different criteria.

The authors suggest that sleep laboratory-based measurement of the actual timing of episodes of DOAs after onset sleep onset - might be a preferred method of determining the likelihood of DOA occurrence for forensic purposes. This is uncertain as the occurrence of LSWS in the sleep laboratory is most often a function of how the sleep study is conducted. Subjects are asked to maintain habitual sleep schedules for a period of time prior to sleep studies. Lights out time and all of the factors that may contribute to First Night Effect common in sleep laboratory studies are likely to effect sleep onset and sleep architecture. In forensic settings it is not possible to recreate or even be sure of the circumstances under which the DOA was thought to have occurred. Prior sleep deprivation is often reported along with situational stress.

Nevertheless, it is clear from this impressive amount of additional data that forensic sleep experts should not assume that SWS will require 30–60 or more minutes to occur following sleep onset for the onset of SWS. Additionally, even in the absence of sleep deprivation the LSWS of uncomplicated, clinically diagnosed patients with a Disorder of Arousal may be as short as 5 minutes after sleep onset.
